# The Molecular Basis of Love

**DOI:** 10.3390/ijms26041533

**Published:** 2025-02-12

**Authors:** Jaroslava Babková, Gabriela Repiská

**Affiliations:** Institute of Physiology, Faculty of Medicine, Comenius University in Bratislava, Sasinkova 2, 81372 Bratislava, Slovakia; jaroslava.babkova@fmed.uniba.sk

**Keywords:** love, behavior, hormones, neuromodulators

## Abstract

Love as a complex interplay of emotions and behaviors is underpinned by an intricate network of neurobiological mechanisms. This review provides insight into the molecular basis of love, focusing on the role of key hormones and neuromodulators. The aim of the paper is to report how these biochemical messengers influence various aspects of love, including attraction, attachment, and long-term bonding. By examining the effects of hormones such as dopamine, oxytocin, vasopressin, and serotonin, we aim to elucidate the intricate relationship between biology and behavior. Additionally, the potential impact of modern lifestyle factors on hormonal balance and their subsequent influence on love and social interactions are outlined. This review provides a useful overview of the molecular underpinnings of love, offering insights into the biological mechanisms that shape human relationships.

## 1. Introduction

Love, as an innate and pervasive force, functions as a universally understood language. It can be viewed as a natural mechanism that ensures the continuation of life. There are several psychological theories that attempt to explain the main components of love [1,2,3]. Despite the advances made in unraveling the meaning of love, it is important to note that love is universal and applies to people of all cultures, races, ethnicities, religions, and sexual orientations and, moreover, love evolves over time. Although passionate love is often presented as an innate human experience grounded in biology, the frequency with which individuals experience it may vary depending on their cultural context, including social structures and belief systems. Therefore, in the field of psychology, questions remain and a framework that can be employed to understand love in all its forms remains to be developed or proposed.

**Figure 1:**
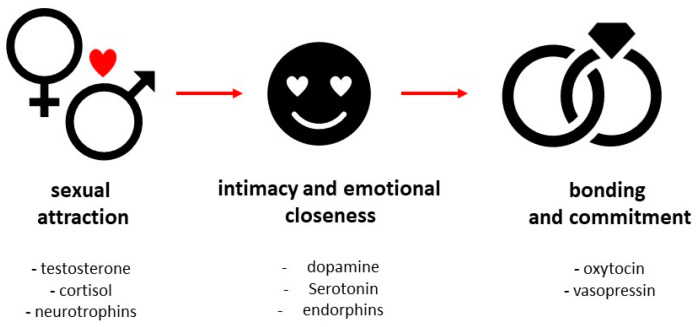
A schematic illustration of the stages of love, including the underlying molecular factors driving human behavior. The initial romantic phase is marked by sexual attraction, mutual appeal, a desire for intimacy, and emotional connection. As the intense chemical reactions wane, a more stable phase develops, characterized by mutual commitment, loyalty, and a long-lasting attachment.

Recently, there has also been growing interest in understanding the biological underpinnings of love. A growing body of research on functional magnetic resonance imaging (fMRI), positron emission tomography (PET), and single photon emission computed photography (SPECT) concluded that there is a specialized network of the brain and certain biomarkers involved in love [4,5,6]. Although brain imaging provides a unique insight into the nature of love, making sense of the psychological significance or inference of fMRI data are problematic [7]. Several endocrine factors were identified to play a role in regulation behavior in various stages of love. However, in many cases, definite proof is still lacking and the few human studies on love are limited by selection bias on the duration of a love affair, gender, and cultural differences [8,9]. The aim of this paper is to propose the review of neuroendocrine factors that represent the molecular basis of behavior and feelings accompanying love and its phases (Figure 1).

Passion, which is a synonym for sexual attraction, is the intense yearning and attraction that define the early phases of a love relationship. Intimacy and emotional bonding are interchangeable terms that convey the degree of interpersonal closeness and emotional connection that people have with one another. Dedication and involvement in the well-being and durability of the relationship are included in commitment, which stands for inclinations toward caregiving [10]. The aim of this review is to provide an overview of how selected hormonal parameters influence our personality features, social behavior, and perceptions of love. The final chapter indicates some adverse effects of modern times on our hormonal milieu that have a negative impact on human interactions. We believe that this paper can provide an interesting overview of the molecular underpinnings of behaviors accompanying love.

## 2. Hormonal Profile of Love

The momentary “madness” of romantic love serves an evolutionary function by forcing people to step outside of their comfort zones and form relationships. Romantic love involves cognitive, emotional, and behavioral components. Cognitively, it is characterized by intrusive thoughts about the partner, idealization, and a strong desire for mutual understanding. Emotionally, it includes feelings of attraction, both romantic and sexual, distress when the relationship is threatened, longing for reciprocation, a desire for deep connection, and physiological arousal. Behaviorally, it manifests as actions like seeking the partner’s attention, studying their behavior, performing acts of service, and maintaining physical proximity. These behaviors are influenced by various identified factors [11].

## 3. Neurotrophins

Neurotrophins, a family of structurally similar proteins, were initially recognized for their crucial role in the survival of sympathetic and sensory neurons. Subsequent research has revealed their involvement in regulating various aspects of neuronal survival, development, formation, and plasticity of neural networks, within both the central and peripheral nervous systems [12]. The neurotrophin family comprises nerve growth factor (NGF), brain-derived neurotrophic factor (BDNF), neurotrophin-3 and neurotrophin-4. One of them playing a role in the early stages of love is NGF. This early phase is also characterized by alterations and circulating levels of NGF from the family of neurotrophins that have been increasingly recognized as potential mediators of anxiety, emotions, and behavioral modifications [13,14]. These molecules were originally described as key regulators of synaptic plasticity and neural survival during development and at adulthood [15]. Interestingly, NGF levels have been shown to be significantly higher in those subjects who had recently fallen in love compared to subjects who were single or engaged in a long-lasting relationship [16]. A positive association between the intensity of early romantic feelings and serum levels of NGF has been identified [17]. Brain-derived neurotrophic factor (BDNF), another member of the neurotrophin family, was not proven to be increased during the early stage of love. However, plasma levels and romantic attachment proved to be related in a sex-specific manner. In fact, women showed a significant and negative correlation between BDNF levels and the avoidance scale [18]. This suggests that BDNF may help to promote social relationships through a specific decrease in avoidance and fear only in women who are naturally more anxious. Increased BDNF signaling was also shown to ameliorate symptoms of depression [19].

## 4. Cortisol

Cortisol, often known as a stress hormone, plays a multifaceted role in human physiology. This key glucocorticoid, produced in the adrenal cortex’s zona fasciculata, is governed by the hypothalamic–pituitary–adrenal axis (HPA). It influences numerous processes in the human body, including stress response mediation, metabolic regulation, modulation of inflammation, and immune system function [20]. The formation of a new romantic relationship is associated with significant alterations in both behavioral and biological stress responses. In the early stages, individuals often exhibit increased focus on subtle nonverbal cues and a heightened fear of rejection, indicating increased stress. However, the establishment of a romantic relationship also requires a certain level of calm and trust. This unique state of arousal is crucial for forming strong bonds and involves a reduction in the stress response. Newly formed romantic relationships (within the first six months) were associated with higher baseline plasma cortisol compared to singlehood or long-term relationships, as reported by Marazziti and Canale (2004) [21]. It is probably a non-specific indicator of some changes that occur during the early phase of a relationship, reflecting the stressful conditions or arousal associated with the initiation of a social connection, which helps to overcome neophobia. Similarly, Loving et al. (2009) reported higher salivary cortisol reactivity in women when asked to think of their loved ones [22]. A more recent study examined the effect of cortisol reactivity to a stressor on self-reported levels of perceived closeness to a stranger. The findings suggested men who exhibited cortisol reactivity reported greater levels of perceived social closeness relative to men who did not experience cortisol elevations [23]. These results highlight the possibility for cortisol fluctuations to underline attachment formation by acting as a catalyst, with high cortisol levels increasing the need and desire for social contact. Contrastingly, Weisman showed that new lovers exhibited lower daily cortisol production measured in saliva samples in comparison with singles, suggesting that satisfying marital relationships reduces (HPA) response [24]. Furthermore, some studies have failed to demonstrate significant variations in cortisol concentrations between women in the early stages of romantic love and single women [25]. Inconsistent findings can be attributed to methodological differences. Firstly, the data obtained from female participants were found to be substantially influenced by the specific phase of the menstrual cycle, as well as interindividual differences. Secondly, diurnal, plasma, salivary, and reactive cortisol measurements have been shown to be unrelated [26]. Thirdly, a potential explanation lies in the specific dimensions of the relationship examined in different studies. Love is considered as a highly specific, goal-directed state that can be very specific and highly variable among individuals. Some studies chose the participant based on the passionate component of love; others include committed love styles. Although the HPA axis is a key factor in bond formation, focusing solely on this system overlooks the complex interplay of neurohormones involved and the influence of other physiological systems. Cortisol is most often conceptualized as a likely biological correlate of neuroticism via activation of the HPA axis. Indeed, higher levels of neuroticism (being moody and worrying) are associated with higher levels of salivary cortisol throughout the day, and elevated cortisol is one of the proposed mechanisms linking neuroticism to poorer health outcomes over time [27,28]. However, numerous other studies have yielded conflicting findings regarding both the strength and direction of this relationship. Therefore, compelling evidence of an association between cortisol levels and neuroticism remains elusive. On the other hand, sensation seekers tend to have lower salivary cortisol levels [29]. The overall body of research examining the link between cortisol and personality traits is relatively small and yields inconclusive results. Meta-analyses suggest a small to negligible effect [30].

## 5. Serotonin

Serotonin (5-hydroxytryptamine, 5-HT) is a bioactive amine exerting pleiotropic effects within both the central nervous system and peripheral tissues. It is primarily recognized for its function as a neurotransmitter/neuromodulator and it is implicated in the physiology of a range of conditions, including mood [31]. Serotonergic mechanisms also mediate the expression of personality traits. Neuroticism, such as anxiety, depression, hopelessness, somatization, guilt, hostility, and affective temperament, have been linked to increased serotonergic signaling [32]. However, to date, there have been no systematic investigations of the role of serotonin activity in the identification and other aspects of personality in other people. On the other hand, it was studied in the context of a wide range of behaviors and experiences, including love. Early romantic love and associated behavior is linked with reduced serotonin levels in the blood. Even though some authors proved this effect only for males [33], individuals with a higher predisposition to fall in love also have lower blood serotonin levels compared to those who have never been in love [34]. Selective serotonin reuptake inhibitors were found to reduce the intensity and duration of feelings of love in a case study of a man who was taking selective serotonin reuptake inhibitors [35]. Individuals in the early stages of romantic love exhibited temporarily similar platelet 5-HT transporter densities to individuals with obsessive–compulsive disorder (OCD). Both groups showed significantly lower densities compared to healthy controls [36]. Serotonin transporter is a membrane protein that transports serotonin from the synaptic cleft back into the presynaptic neuron; a reduced transporter density implies that an increased number of serotonin molecules would be present in synapses. This suggests potential shared neurochemical alterations within the serotonin (5-HT) system, particularly related to the obsessive component, intense preoccupation, and anticipation common to both conditions [36]. The prefrontal serotonergic projection and the serotonin 2 receptor seems to be associated with the manic, stalking aspect of romantic love [37]. The question of whether central serotonin levels are genuinely diminished remains unresolved.

Genetic components affecting serotonin signaling are emerging as additional factors influencing behavior in romantic relationships. The G allele of the C-1019G (rs6295) polymorphism leads to a higher expression of the *5-HT1A* gene (encodes a G protein-coupled receptor for 5-hydroxytryptamine (serotonin)). It was shown that individuals with the CG/GG genotype reported greater difficulty in identifying their own feelings and seemed to be less comfortable with having close relationships to others than individuals with the CC genotype [38]. A study on 579 Chinese undergraduate students showed that individuals carrying the G allele (CG/GG) of C-1019G polymorphism were more likely to be single than CC carriers [39].

## 6. Dopamine

Dopamine, the primary catecholamine neurotransmitter in the human central nervous system, plays a key role in regulating diverse functions such as cognition, emotions, movement, appetite, and endocrine activity. Beyond the central nervous system, dopamine also influences peripheral functions, including cardiovascular and renal activity, gastrointestinal motility, and the endocrine system [40]. Higher dopamine levels are often associated with increased reward sensitivity, novelty seeking, and social engagement [41]. Studies have shown that individuals with higher levels of dopamine receptor availability tend to score higher on measures of extraversion, indicating a greater propensity for social interaction, excitement-seeking, and positive emotions [42]. When experiencing love, dopamine activates the reward circuit, contributing to the pleasurable experience, which can be likened to the euphoria associated with cocaine or alcohol use. This suggests that romantic love engages the same brain regions implicated in addiction. The reward center can be activated through various means [43,44]. Data from animal research also prove that dopamine release is not only important for rewarding feelings but also for the mate preference. When a monogamous female prairie vole is mated with a male, she forms a distinct preference for this partner; however, when a dopamine agonist is infused into the nucleus accumbens, she begins to prefer a male present at the time of infusion, even if she has not mated with this male. Furthermore, a striatal output region through the ventral pallidum is strongly implicated as critical to male prairie vole mate preference behaviors [45]. Studies in humans that are intensively “in love” proved the activation of specific dopamine-rich areas associated with mammalian reward and motivation [46]. It is noteworthy that these regions differ from those responsible for sex drive, indicating that dopamine can be considered as an initial driving force and arousal component motivating individuals to make a mate choice. It can be hypothesized that it helps to discriminate among potential mating partners and focus courtship activities on particular individuals to save time and energy [47].

## 7. Endorphins

Endorphins are opioid neuropeptides acting as both neurotransmitters or neuromodulators within the central nervous system and as hormones when secreted by the pituitary gland [48]. Beta-endorphins are the most extensively researched and abundant of the three known endorphin types, and its properties largely define our general understanding of endorphin function as a whole. It is originated by processing enzymatic cleavage of pro-opiomelanocortin (POMC) cells primarily located in the hypothalamus [49]. Beta-endorphins are well-known for their powerful pain-relieving properties; they also play a role in boosting confidence, enabling control of emotions, generating feelings of euphoria, and the maintenance of homeostasis [50]. While dopamine is often highlighted in discussions of reward systems, beta-endorphins also play a role in these pathways [51]. Beta-endorphins also seem to modulate serotonin activity. It has been suggested that a mild increase in the beta-endorphin level creates a sense of well-being and euphoria often presented at early stages of love [52]. Beta-endorphins, acting in concert with other factors, are implicated in the motivational and precopulatory phases of sexual behavior [53]. Although some research indicates that increased endorphin levels during sexual activity in humans may contribute to pair bonding and attachment, contradictory findings challenge a definitive relationship between sexuality and beta-endorphin concentrations [54]. A small study of 10 healthy women found no significant changes in plasma endorphin levels associated with sexual arousal or orgasm [55]. While the role of endogenous opioids in human sexual function and behavior is not fully understood, it is established that exogenous opiates have detrimental reversible effects on the sexuality of both males and females. These effects include diminished sexual desire, impaired arousal, reduced genital response, delayed or absent ejaculation, orgasmic dysfunction, and infertility [56]. One study investigated the effects of naloxone, an opioid antagonist, on female sexual response. The researchers found that administering naloxone in two separate doses of 2 mg each time led to an increase in the intensity of orgasms and the overall pleasure experienced by the women. This suggests that blocking opioid receptors, which are typically activated by endorphins, can paradoxically enhance certain aspects of female sexual experience. However, the same dose of naloxone (2 mg) administered only once had the opposite effect, inhibiting both sexual arousal and the ability to achieve orgasm. This indicates the dual nature of endogenous opioids, including beta-endorphins, which can have both inhibitory and excitatory actions. However, the mechanisms responsible for this dose-dependent effect are not yet fully understood [57]. Current understandings of the mechanisms through which endorphins regulate human sexuality and behavior are also limited.

## 8. Testosterone

Testosterone is a steroid sex hormone with an important role in the physiology of both sexes. It is mostly known for its role in the development of sex organs and physical maturation during puberty. In men, the main production of testosterone is localized to the smooth endoplasmic reticulum of Leydig cells in the testicles. Half of the testosterone amount in females is generated by the ovaries; the rest by the cortex of suprarenal glands [58]. Sex hormones exert variable influence on personality and also on behavior during romantic attachment. Salivary testosterone has been found to be associated with extraversion-related traits such as sensation seeking [59]. Likewise, testosterone and extraversion are both associated with dominance tendencies in mating contexts. For instance, one particular study demonstrated that higher testosterone levels were associated with dominant behavior in men towards other men, as well as a dislike for male competitors. Additionally, women self-reported a higher degree of connection with men exhibiting higher testosterone levels [60]. However, reliable links between extraversion and testosterone are not always found [61]. Higher salivary testosterone was associated with a facet of lower conscientiousness but not consistently [62,63]. Testosterone appears to be largely unrelated to openness to experience. There is evidence that testosterone measured in saliva is negatively associated with anxiety, stress, and depression, all of which are factors of neuroticism [64]. Even though the results from multi-level models and meta-analyses concluded mostly weak, non-significant associations between testosterone and personality traits, there is a need to discuss and examine heterogeneity in hormone-personality associations [30].

**Figure 2:**
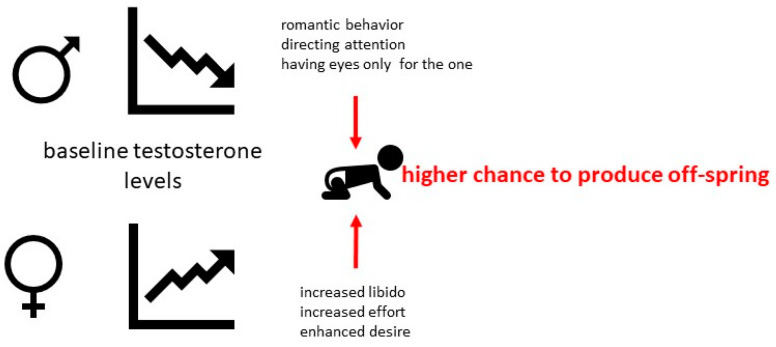
Sex-specific dynamics of baseline testosterone in the early stage of a relationship. It involves a decrease in testosterone levels among men, potentially leading to more focused sexual attention primarily directed towards their romantic partner. Conversely, an increase in testosterone levels in women might enhance their sexual desire and proceptivity. This hypothetically contributes to maximizing the likelihood of reproduction.

Despite extensive research the precise nature of sex hormone fluctuations, their impact on our behavior during love remains elusive, as existing research has produced inconsistent findings. In a study encompassing 24 participants, Marazziti et al. revealed that men in the nascent stages of romantic love (within the initial six months of a relationship) exhibited markedly decreased levels of the follicle-stimulating hormone (FSH) and testosterone in blood serum when compared to both single individuals and those engaged in long-term, stable partnerships. In contrast, newly in-love women displayed significantly elevated testosterone concentrations relative to control subjects [21]. The observed phenomenon could be hypothetically attributed to the hormonal changes associated with early-stage romantic love. Specifically, the reduction in testosterone levels among men may contribute to a narrowed focus of sexual attention, directing it primarily towards the romantic partner. In contrast, the elevation of testosterone in women may enhance sexual desire and proceptivity, potentially resulting in a convergence of hormonal profiles and sexual behaviors between the two sexes (Figure 2).

Conversely, a subsequent study involving female participants yielded divergent results. These findings indicated that women in the early stages of romantic love exhibited lower blood serum testosterone concentrations relative to their single counterparts [25]. The lack of consistency in the findings can be attributed to variations in research methodologies and also age or cross-cultural differences. Specifically, when measuring testosterone in women, results are significantly impacted by many crucial factors; for instance, the stage of a romantic relationship, the amount of sexual activity [65], the menstrual cycle phase or menopause [66], and whether the female participant is in a relationship with a male partner who is geographically proximate or distant. Interestingly, female participants romantically involved with partners residing in the same city exhibited significantly lower testosterone concentrations than those in long-distance relationships [67]. This finding highlights an additional methodological challenge in such studies. The presence of a partner significantly impacts hormone levels. Moreover, the couple’s cohabitation status (whether they live together or are casually dating) is a crucial variable that complicates the direct correlation between testosterone concentrations and romantic behavior.

Several studies have tested the reactive testosterone response to a stimulus that appears to be unrelated to baseline testosterone concentrations. Results from a human population of young women demonstrated a reactive increase in salivary testosterone after watching a video depicting intercourse between an attractive man and woman, compared to women who watched neutral videos [68]. Consistently, studies have demonstrated that interactions with young women elicit an increase in salivary testosterone levels also in men, regardless of whether the interaction occurred in a laboratory or a natural environment. Interestingly, experiments conducted with a Dominican tribe revealed that men experienced an increase in salivary testosterone levels following interactions with women, but only when the woman was single and not already partnered with another man in the tribe. They had lower salivary testosterone if the woman was a conjugal partner of a close friend [69]. Based on these observations, it can be hypothesized that a positive testosterone surge mirrors an internal decision to initiate mating behavior. In contrast, men and women often have lower testosterone when they are partnered parents [70].

It is also evident that the concentration of testosterone, whether it is at the current level or that experienced during fetal development, plays a role in determining the specific mating strategies and types of romantic behavior individuals employ. Our previous work showed that plasma testosterone levels in young men negatively correlated with a romantic loving style and selfless altruistic love [71]. Moreover, not only does actual plasma matter, but also prenatal androgen priming can play a role. Prenatal testosterone exposure cannot be directly measured but can be estimated by the 2D:4D ratio. This is the ratio of the length of the index finger to the ring finger. Some studies suggested and proved that a higher 2D:4D ratio in men might be linked to lower prenatal testosterone exposure [72]. Men with lower prenatal testosterone exposure may not naturally exhibit the same level of typical masculine traits and dominance compared to men with higher prenatal testosterone exposure [73]. To succeed in relationships, these men might compensate for lower displays of traditional masculinity by practicing different styles of love compared to men with higher prenatal testosterone. They tend to prioritize nurturing and playful interaction, keeping the relationship fun and exciting through games, humor, and shared experiences [71]. However, it is noteworthy here that the use of digit ratios as indicators of prenatal androgen exposure in adults is a subject of ongoing debate and remains controversial. In conclusion, while the complex interplay between testosterone and romantic relationships is not fully understood, it is clear that testosterone plays a role in shaping our romantic behavior and is also influenced by the type of behavior we exhibit.

## 9. Oxytocin and Vasopressin

Oxytocin and vasopressin are closely related peptide hormones consisting of nine amino acids. They are synthesized primarily in the hypothalamus and released from the posterior pituitary gland. Oxytocin was first described for its important role in stimulating uterine contractions and milk ejection after birth. Vasopressin was recognized as vital for water balance, primarily through its regulation of urine concentration within the kidneys [74].

Oxytocin has a fundamental role in social behavior, causing one to be open, extrovert, and agreeable. In humans, supporting evidence shows that oxytocin enhances our ability to trust or affiliate with others [75]. It is important to also consider here that personality traits may influence oxytocin levels, and conversely, oxytocin may shape personality development. The initial phase of romantic love, characterized by infatuation and mutual attraction, typically transitions into a more stable, secure attachment style marked by loyalty and secure partnership [76]. Based on a large body of research, oxytocin and vasopressin can be argued as biological correlates during this phase [77]. This was widely proved in animal studies comparing socially monogamous and non-monogamous vole species. They highlighted the differences in oxytocin and vasopressin receptor expression within brain regions crucial for social attachment. The higher oxytocin receptor density in the brains of monogamous male voles predicts different mating tactics, mating success, and social monogamy [78,79]. In monogamous prairie voles, levels of vasopressin receptors (encoded by the gene *AVPR1*) in specific brain regions also predict fidelity and pair-bonding behavior [80]. Pharmacological studies have demonstrated that exogenous oxytocin administration is sufficient to induce pair-bonding behavior in voles, while the administration of oxytocin receptor antagonists disrupts these behaviors [81]. A recent study from 2023 unexpectedly neglected previously published findings. Genetic *Oxtr*-null mutant prairie vole lines revealed that social bonding can occur without oxytocin signaling [82]. This contradictory finding leaves the question of how precisely oxytocin and vasopressin regulate open social attachments. It also highlights the need for a cautious interpretation of data and for a refined understanding of the molecular pathways underlying social attachment behaviors.

It has been argued that the prairie vole pair-bonding neural circuit may serve as a model for understanding human mechanisms of attachment and love. Building upon findings from animal model experiments, a series of human studies has demonstrated the influential role of oxytocin and its concentrations on the perception and experience of love and romantic relationships. A study of 129 adults correlated circulating oxytocin concentrations with the perception of one’s partner and proved the role of this peptide in social bonding. Results showed that higher circulating oxytocin concentrations were associated with a greater ability to attenuate negative expressive behavior towards one’s partner, a greater tendency to overlook negatives, and a higher appreciation of gratitude for their presence. Oxytocin, in this case, functioned as rose-colored glasses, helping individuals focus on their partner’s positive attributes and appreciate them [83]. Another study on 34 young married couples showed that higher concentrations in salivary and plasma oxytocin are linked with lower distress and better quality of marriage [84]. Higher peripheral plasma oxytocin concentrations were measured in those couples exhibiting mutual support (based on self-report), warm contact, physical intimacy, massages, proximity, and frequent hugging [85]. Results from the intervention study demonstrated that increasing intimacy and warm contact over a four-week period led to a significant increase in salivary oxytocin concentrations in both men and women compared to the control group [86]. Increased oxytocin levels in both men and women have been shown to improve not only relationship quality and bonding but also overall health and immune function [87,88,89]. It is noteworthy here that a variety of individual and contextual factors such as sex, social context, and psychiatric history may influence oxytocin levels, the effect on certain behavior, and also the response of oxytocin to certain stimuli. According to some other studies, oxytocin does not necessarily need to be linked with positive and improved social behavior. Individuals reporting higher stress in relationship or increased depression symptoms can also display higher oxytocin levels [90,91]. Research indicates that individuals with psychiatric diagnoses or trait characteristics, including depression, borderline personality disorder, or high trait aggression, may be susceptible to adverse social or emotional consequences following oxytocin administration [92]. Some studies also have found that women and men can have sex-specific responses to oxytocin as well [93,94].

Much less is known about circulating levels of vasopressin and its effect on social behavior. There are some studies investigating the effect of genetic background. Individual variability in expression levels is associated with individual variation in polymorphic microsatellite lengths in the 5′ regulatory region of the prairie vole *AVPR1* gene for vasopressin receptors [95]. Such a gene regulatory mechanism may occur in humans as well. Humans have three microsatellite polymorphisms in the 5′ regulatory region of the *AVPR1* gene [96]. These microsatellites influence location and receptor density in some brain areas, with behavioral consequences in both voles and humans. For instance, shorter repeats in RS3 polymorphism are linked with decreased relative promoter activity. Males carrying shorter alleles reported lower social bonding, more frequent crises, lower quality of marriage, and perceived problems in partnership. Compared to men with increased vasopressin receptor expression, they exhibited a lower rate of marriage [97]. A series of studies involving exogenous peptide administration has also provided evidence that oxytocin and vasopressin modulate social behavior in relationships. Oxytocin directly enhances monogamous behavior and fidelity in men by modifying their behavior. Exogenous administration of oxytocin to men in monogamous relationships caused them to keep a greater distance from other attractive female strangers [98]. The intranasal administration of oxytocin could enhance trust behavior [99].

While the interpretation of results requires caution due to existing uncertainties and open questions, the overall evidence points to oxytocin and vasopressin as important biological factors contributing to bonding and attachment in relationships (Table 1).

## 10. Side Effects of the Modern World

Modern times introduce numerous specific factors that significantly impact our lives, from our physical health to our emotional well-being and our processing of love. For instance, studies conducted on American population have shown longitudinal declines in testosterone levels independent of chronological aging due to various reasons such as higher body mass index (BMI), decreased physical activity, environmental factors, etc. [120]. Testosterone levels in men today are statistically lower than those of men from 20 years ago and this trend was also detected in young adults and adolescents [121]. This fact can be associated with various comorbidities, but also with the possibility that a decrease in testosterone levels could influence men’s interactions with the opposite sex and decrease libido. Based on data from the American General Social Survey, American adults had sex about nine fewer times per year in the early 2010s compared to the late 1990s [122]. Data from other countries and cultures are currently unavailable. Future research will be essential to investigate how declining testosterone levels impact the behavior of younger generations and what the consequences might be

Current research is increasingly examining the psychological effects of social media use. There is now a consensus that excessive social media consumption can lead to significant behavioral, neurological, and psychological changes. Moreover, it can alter our capacity to experience love, physical intimacy, and genuine human connection. Every notification on a mobile device triggers a surge in dopamine concentration. Dopamine levels then rapidly decline, prompting the brain to seek this reward again. Consequently, the individual experiences a recurring urge to check their phone screen [123]. Social media access is readily available and free. The dopamine hit from social media is easier and more readily available than that of human interaction, making it a more appealing option and, thus, diminishing the need for face-to-face interactions. Supporting this notion, research conducted in Germany analyzed the daily needs of 7827 individuals aged 18–85. The study identified social media as a particularly weak link in terms of resisting habitual behaviors [124].

Many studies show that one of the most common consequences of social media addiction is the relationship problem. There is a growing concern regarding the decline of long-term marriages in recent years. This decline may be partly attributed to the rise in social media, which has facilitated the formation of online emotional connections and interactions with geographically distant individuals, 24 h a day, 7 days a week. Social media platforms provide a space for individuals to engage in conversations with anonymous online companions on topics that might be considered taboo or difficult to discuss with close family members or spouses. These conversations can quickly escalate, potentially leading to the sharing of sexual fantasies. This phenomenon, known as “virtual infidelity”, can erode trust within marriages and contribute to their eventual breakdown [125]. Intensive use of social media can have damaging effects on human relationships and should be used with caution.

## 11. Conclusions

Love, a fundamental human experience, has captivated philosophers and scientists for centuries. By investigating the neurobiological and hormonal underpinnings of love, we can gain valuable insights into its evolutionary origins and its role in shaping human behavior. Love and attachment play crucial roles in human health and well-being. Studying the neurobiology of love can lead to novel treatments for conditions like loneliness, depression, and anxiety disorders. Understanding the hormonal mechanisms underlying love can also inform the development of new therapies for couples experiencing relationship difficulties. An overview of the love-related molecules discussed is presented in Table 1.

Future research should focus not only on the role of specific neurotransmitters, hormones, and genetic factors, but also on the impact of cultural factors on the expression of love and particularly biological responses. There is very limited data available. We found a study indicating that individuals from Western cultures tend to favor verbal communication in expressing romantic affection, whereas East Asians are more inclined to use gift-giving as a means of conveying their romantic feelings [126]. Research in 45 countries showed that higher levels of modernization is associated with higher mean levels of love, especially intimacy. This lends partial support to the idea that modernization processes can influence how love is experienced [127]. However, these works examined the influence of cultural aspects on the expression and experience of love, rather than biological responses. Thus, research investigating the influence of culture and society on biological responses and hormonal fluctuations is lacking. Future studies should address this gap. Future research should also focus on longitudinal monitoring of how modern lifestyle influence our hormonal environment and how this is reflected in our behavior and experience of love. Such research will contribute to our understanding of the human condition and inform the development of interventions to promote healthier and more fulfilling relationships.

However, it is important to acknowledge again that love is an extremely complex phenomenon, varying greatly from person to person and manifesting in diverse forms and trajectories. Moreover, significant disparities exist between the phenomenological experiences of falling in love, established romantic love, and sexual activity. These disparities are amplified when considering initial versus repeated occurrences of romantic love. Hormonal influences may exhibit differential effects across these experiences. Furthermore, a multitude of factors can also contribute to variability, including subject age, personality, cultural background, religious beliefs, residential environment (urban vs. suburban), geographical location, and socio-economic status. To accurately characterize, describe, and comprehend love and all its stages in every individual, a substantial number of well-designed detailed studies would be required. This remains a crucial limitation in this field and presented data must be interpreted with caution. Furthermore, it must be emphasized that a host of neural systems, neurotransmitters, neuromodulators, endogenous opioids, sex steroids, molecules of the HPA axis, and peptides interact with each other, influencing one another, and are part of a larger physiological equation. Moreover, the modern world presents numerous challenges that must be considered, and strategies to mitigate their adverse effects should be implemented.

## Figures and Tables

**Table 1:** Summary of love-related molecules.

Love-Related Molecule	Psychological Effect	Brain Region	Receptor/Interacting Molecule	References
NGF, BDNF (peptides)	Social interaction, anxiety, behavioral, and emotional modifications	Hippocampus, hypothalamus, cortex	p75NTR TrkA, TrkB, TrkC	[12,100]
Cortisol (steroid)	Decision making, arousal, initiation of contact, response to stress	Amygdala, hippocampus, and prefrontal cortex, brainstem nuclei	GR-NR3C1 MR	[101]
Serotonin (monoamine)	Emotions and feelings processing, stalking and obsessive component of romantic love, preoccupation, anticipation, mood regulations	The raphe nuclei in brain stem, projection sites to the hippocampus, amygdala, and cortex.	5-HT1, 2, 4 G coupled receptor 5-HT3 receptor a ligand gated ion channel	[37,102]
Dopamine (monoamine)	Desire, reward, sensation seeking, motivated behavior, feelings of pleasure, mate preference, expectation, representation of goals, and integration of sensory inputs	The substantia nigra pars compacta and the ventral tegmental area, projections make up the nigrostriatal, mesocortical and mesolimbic pathways	D1–D5 G coupled receptors	[103,104,105,106]
Endorphins (peptides)	States of pleasure, sex, love, motivational and pre-mating stages of sexual behavior, enhance well-being	Cerebral cortex, brainstem, caudate nucleus, thalamus-midbrain, hippocampus, amygdala	Mu-receptors-primarily delta-receptors, kappa-receptors, nociceptin receptors	[48,107]
Testosterone (steroid)	Emotional recognition and expression	Amygdala, inferior frontal gyrus	AR-NR3C4	[108]
	Response to sexual stimuli	Hippocampus, frontal gyrus		[109]
	Decision making	Orbitofrontal cortex		[110]
	Facial expressions	Hippocampus, amygdala		[4,111]
Oxytocin, vasopressin (peptides)	Empathy for facial expressions	Amygdala, insula	OR V1a, V1b V2 (not in brain) G coupled receptor	[112]
	Social attachment, mating strategies towards monogamy, appreciation and gratitude of the partner	Prefrontal cortex, left and right precuneus, nucleus accumbens, amygdala, prelimbic cortex		[113,114]
	Trust, trustworthiness	Amygdala, midbrain, dorsal striatum, insula		[115]
	Subjective feelings	Insula		[116]
	Emotional patch	Anterior cingulate cortex, anterior insula		[117]
	Compensatory adjustments in cognitive control	Anterior cingulate cortex		[118]
	States of happiness, interoception	Anterior cingulate cortex		[103]
	Social interactions that involve assessing of emotions	Anterior cingulate cortex		[103]
	Pleasurable feelings and pair-bonding	Ventral tegmental area		[7,119]

NGF = nerve growth factor; BDNF = brain-derived neurotrophic factor; GR = glucocorticoid receptor; NR3C1 = nuclear receptor subfamily 3, group C, member 1; MR = mineralocorticoid receptor; D1–5 = dopamine receptor 1–5; p75NTR = neurotrophin receptor; TrkA, TrkB, TrkC = receptor tyrosine kinases A, B, C; AR = androgen receptor; NR3C4 = nuclear receptor subfamily 3, group C, member 4; 5-HT1–4 = postsynaptic serotonin receptor; OR = oxytocin receptor.
